# Comparative Metabolomics Reveals Two Metabolic Modules Affecting Seed Germination in Rice (*Oryza sativa*)

**DOI:** 10.3390/metabo11120880

**Published:** 2021-12-17

**Authors:** Hao Guo, Yuanyuan Lyv, Weikang Zheng, Chenkun Yang, Yufei Li, Xuyang Wang, Ridong Chen, Chao Wang, Jie Luo, Lianghuan Qu

**Affiliations:** 1 National Key Laboratory of Crop Genetic Improvement and National Center of Plant Gene Research (Wuhan), Huazhong Agricultural University, Wuhan 430070, China; haoguo@hainanu.edu.cn (H.G.); lvyuanyuan@hainanu.edu.cn (Y.L.); zhengweikang@webmail.hzau.edu.cn (W.Z.); victoryang@webmail.hzau.edu.cn (C.Y.); yf.li@webmail.hzau.edu.cn (Y.L.); 18437959097@163.com (X.W.); jie.luo@mail.hzau.edu.cn (J.L.); 2 College of Life Science and Technology, Huazhong Agricultural University, Wuhan 430070, China; 3 College of Tropical Crops, Hainan University, Haikou 570228, China; ridong.chen@hainanu.edu.cn (R.C.); chaowang@hainanu.edu.cn (C.W.); 4 College of Horticulture and Forestry Sciences, Huazhong Agricultural University, Wuhan 430070, China

**Keywords:** rice, seed germination, metabolome, metabolic module, shikimic acid

## Abstract

The process of seed germination is crucial not only for the completion of the plant life cycle but also for agricultural production and food chemistry; however, the underlying metabolic regulation mechanism involved in this process is still far from being clearly revealed. In this study, one *indica* variety (*Zhenshan 97*, with rapid germination) and one *japonica* variety (*Nipponbare*, with slow germination) in rice were used for in-depth analysis of the metabolome at different germination stages (0, 3, 6, 9, 12, 24, 36, and 48 h after imbibition, HAI) and exploration of key metabolites/metabolic pathways. In total, 380 annotated metabolites were analyzed by using a high-performance liquid chromatography (HPLC)-based targeted method combined with a nontargeted metabolic profiling method. By using bioinformatics and statistical methods, the dynamic changes in metabolites during germination in the two varieties were compared. Through correlation analysis, coefficient of variation analysis and differential accumulation analysis, 74 candidate metabolites that may be closely related to seed germination were finally screened. Among these candidates, 29 members belong to the ornithine–asparagine–polyamine module and the shikimic acid–tyrosine–tryptamine–phenylalanine–flavonoid module. As the core member of the second module, shikimic acid’s function in the promotion of seed germination was confirmed by exogenous treatment. These results told that nitrogen flow and antioxidation/defense responses are potentially crucial for germinating seeds and seedlings. It deepens our understanding of the metabolic regulation mechanism of seed germination and points out the direction for our future research.

## 1. Introduction

Successful germination of seeds and the normal development of seedlings are decisive factors in the reproduction of seed plants [1,2]. In agriculture, seed germination is regarded as the starting point and is closely related to grain yield [3], and in food chemistry, germination treatment is a common way to improve seed nutrition [4]. Therefore, it is of great theoretical and practical significance to reveal the mechanism of seed germination. According to the classical definition, seed germination encompasses events from imbibition to radicle protrusion through seed coverings [5]. This process is relatively short in the plant life cycle, but the underlying mechanism is complicated. As life science studies have progressed, researchers have revealed the mechanism of seed germination from many aspects, including hormone regulation and interaction, iron balance, amino acid residue repair, DNA damage repair, and the identification of many related genes, which have greatly deepened our understanding of this process [5,6,7,8,9,10]. However, the mechanism of seed germination is still far from being fully revealed.

Physiologically, seed germination is characterized by a transition from a quiescent to a highly active metabolic state, coupled with the triphasic uptake of water [11,12]. In this process, both the decomposition and synthesis of metabolites and the conversion and consumption of energy occur; thus, a variety of metabolites are produced [4,8,13]. Some of these metabolites have been clearly found to regulate seed germination. The typical representative is phytohormones, whose involvement in seed dormancy and germination has always been a popular topic in seed physiology and biochemistry research [7]. The most well-known phytohormone, abscisic acid (ABA), can inhibit the process of seed germination. At the same time, complex interactions exist between ABA and other phytohormones. Gibberellin (GA) is known to be the primary phytohormone that acts antagonistically with ABA and can promote seed germination [14], while jasmonic acid (JA) shows a synergetic effect with ABA in regulating seed germination [15]. Exogenous treatment with another phytohormone, auxin, was found to repress soybean seed germination by decreasing the GA/ABA ratio [16]. Therefore, the functions of various phytohormones during seed germination are complex and subtle. Polyamine is also a key modulator of plant growth and development, and its involvement in seed germination has attracted research attention in recent years. Germination led to the accumulation of the total polyamine content in seeds [17]. Polyamine was even found to be distributed compartmentally in germinating seeds, which may suggest its relationship with this process [18]; presoaking with polyamine was found to improve seed germination [19]. This effect may involve starch metabolism, phytohormone interactions, antioxidant defenses, and relevant gene expression [20,21]. Therefore, the potential applications of polyamine in agriculture and plant biotechnology have been raised in recent years [22]. In addition, the involvement of amino acids in seed germination is also worthy of attention. It was found that essential amino acids such as lysine, methionine, leucine, isoleucine, threonine, phenylalanine, and valine increase significantly during germination in wheat, brown rice, and triticale [23]. In addition, inhibiting valine degradation can influence the development and germination of Arabidopsis seeds [24]. Asparagine, arginine, and γ–aminobutyric acid (GABA) are the three most common amino compounds involved in the mobilization of nitrogen reserves in germinating chestnut seeds [25]. Therefore, as the major transport form of nitrogen and the key factor of the carbon/nitrogen balance in plants, the role of amino acids in seed germination should not be ignored. These reports have laid a solid foundation for revealing more metabolites that can regulate seed germination and elucidating the mechanism of this process.

With the development of detection techniques, the establishment and application of metabolomics provide a good opportunity to investigate seed germination. Due to its high throughput and efficiency, metabolomics has been applied to research on the mechanism of seed germination in several common crops, including barley, tomato, and mung bean [26,27,28]. Rice is one of the most important food crops, with half of the world’s population depending on it as a staple. Many researchers have devoted themselves to revealing the mechanism of rice seed germination by various means, including metabolomics. Shu et al. (2008) explored the metabolite profiling of germinated seeds (0, 24, 48, 72, and 96 h after imbibition) in three rice materials, thus pioneering metabolomics research on seed germination in rice [29]. Transcript and metabolite profiling were performed on rice embryo tissue samples collected at various time points during germination (0, 1, 3, 12, 24, and 48 h after imbibition), thus forming a foundation for examining transcriptional and posttranscriptional processes during germination [30]. Ten years later, there have been an increasing number of studies on the metabolomics of seed germination in rice. The rice varieties *02428* (*japonica*) and *YZX* (*indica*) were used to study the metabolic profile during germination under low temperature, and the metabolome was detected at different time points during germination (0, 2, 3, and 4 days after imbibition) to screen for key metabolites involved [31]. To reveal the regulatory mechanism of the differences in growth rate between the two varieties (one *indica* and one *japonica*) during germination and young seedling growth, seeds at 0, 2, 3, and 4 days after imbibition were examined on a large scale by using RNA sequencing and a widely targeted metabolomics method [32]. The changes in the metabolite profiles during germination (0, 4, 8, 12, 16, 24, 32, 40, and 48 h) in one variety were analyzed, and carbohydrate metabolism, the tricarboxylic acid (TCA) cycle, and lipid metabolism were determined to be the main processes that changed during the germination of brown rice [33].

Undoubtedly, these studies have greatly deepened our understanding of seed germination metabolism in rice. However, at the same time, we can see that the current research on the metabolome during seed germination is still limited; much of this research has examined germinating/germinated samples with intervals of days after imbibition and mainly focuses on early seedling development but pays insufficient attention to comparative metabolomics between varieties at the early germination stage. Therefore, in this study, by using two varieties with different germination speeds, we detected the metabolome of seeds at different time points, mainly in the early germination stage (0, 3, 6, 9, 12, 24, 36, and 48 h after imbibition). Based on a widely targeted metabolomics method and a comparative study, a set of key metabolites and two metabolic modules regulating seed germination in rice were acquired.

## 2. Results

### 2.1. Basic Germination Characteristics of ZS97 and NIP

**Figure 1:**
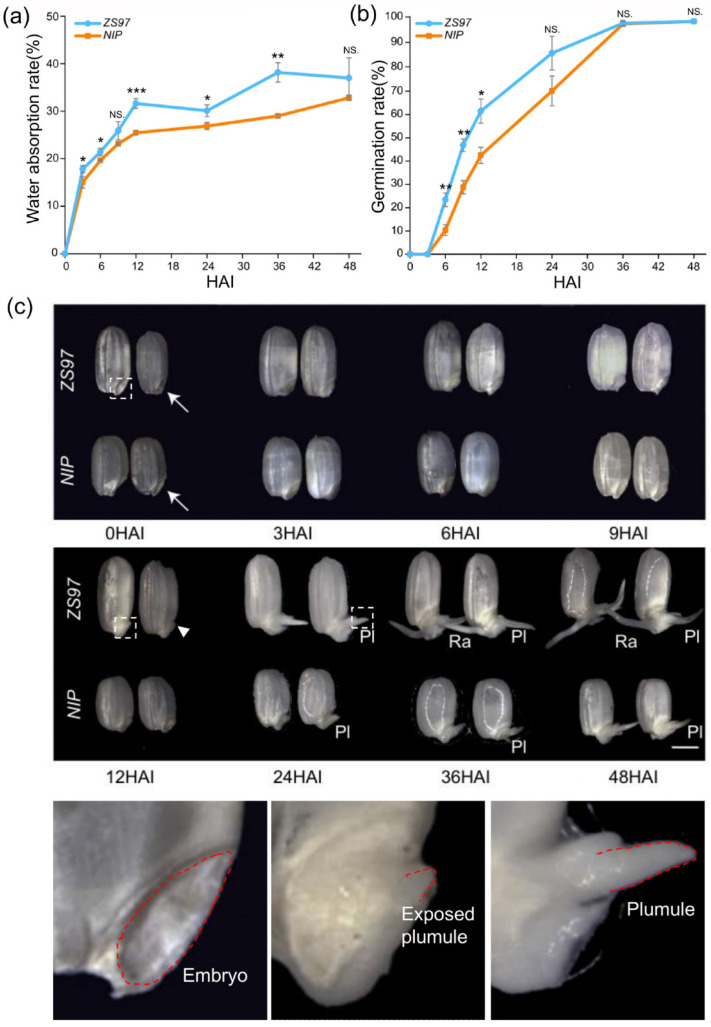
Basic characteristics of seed germination in *ZS97* and *NIP* at different stages. (**a**) Water absorption rate. (**b**) Germination rate. Results of Student’s *t*-test (compare between *ZS97* and *NIP* at the same time point): * *p* < 0.05; ** *p* < 0.01; *** *p* < 0.001. NS. no significant difference. (**c**) Morphological changes in seeds during the germination process. The typical embryo, exposed plumule and developed plumule were amplified at the bottom. Different time points were shown as HAI (hours after imbibition). The embryo is indicated by the arrow. The emergence of plumule is indicated by the arrowhead. Pl, plumule; Ra, radicle. The bar is shown as 2 mm. *ZS97*, *Zhenshan97*. *NIP*, *Nipponbare*.

Two varieties were used for analysis in this study. One was the *indica* variety *ZS97*, and the other was the *japonica* variety *NIP*, with the former germinating faster than the latter. First, detailed characteristics of germination were analyzed, including the water absorption rate and germination rate at eight time-points (0, 3, 6, 9, 12, 24, 36, and 48 HAI). As well known, the uptake of water by seeds is triphasic [6,12,34]. It begins with a rapid initial uptake without visible morphological changes (Phase I, imbibition). Phase I is followed by a plateau phase (Phase II) in which the water content is constant but metabolic activity increases. Phase II then ends with radicle protrusion and is followed by a further increase in water uptake (Phase III) with axis elongation and the establishment of a young seedling. Overall, the trend of water absorption rates at the eight time-points for the two varieties in our study is in accordance with the well-known three-phase theory (Figure 1a). In rice, Phase I is approximately from 0 to 12 HAI, and Phase II is from 12 to 36 HAI. Compared with that of *ZS97*, the germination of *NIP* was slower in terms of both the water absorption rate and germination rate (Figure 1a,b). In addition, observation of morphological changes during germination also showed that seeds of *ZS97* germinated faster than *NIP*. It was often the plumule that protrudes first, instead of the radicle, during seed germination in both varieties of rice. At 12 HAI, the embryo of *ZS97* showed significant signs of plumule protrusion (Figure 1c, arrowhead), while the embryo of *NIP* did not. At 24 HAI, the protruding plumule of *ZS97* was obviously longer than that of *NIP* (Figure 1c, Pl). This superiority of *ZS97* was maintained across phases, even when the radicle protruded (Figure 1c, Ra).

### 2.2. Metabolite Profiling in the Germination Process of ZS97 and NIP

To explore the mechanism behind the differences between the two varieties in terms of germination speed, we conducted a comprehensive study on the metabolome of seeds at eight time-points. The metabolome of all samples was detected by a widely targeted metabolomics method based on HPLC–MS/MS. A total of 380 annotated metabolites were detected for further analysis (Supplementary Tables S1 and S2). Among them, the most numerous metabolites were lipids (96 in total), followed by flavonoids (70 flavonoids and their derivatives), and amino acids and their derivatives (66 in total). In addition, vitamins (23 including choline erucate and nicotinamide) and phytohormones (13 including jasmonic acid and abscisic acid) were also detected. For each variety, three replicates were set up at one time-point and the data of metabolome with 48 samples was acquired (Table S2). Among these samples, one replicate of ZS97-24HAI (ZS-24H-1) was quite different from the other two and was then eliminated in the subsequent analysis. 

**Figure 2:**
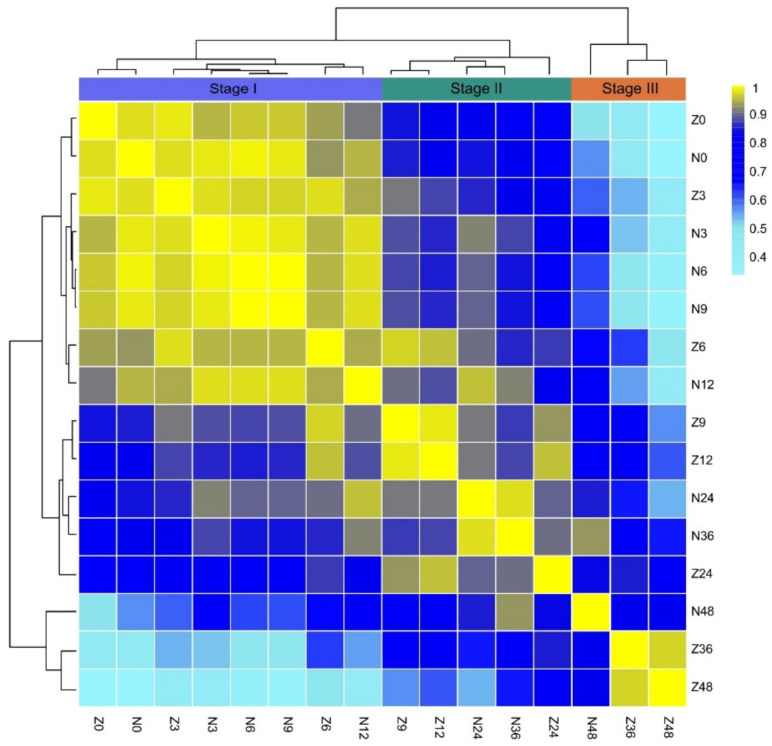
Correlation between the metabolomes of different stages of seed germination in *ZS97* and *NIP*. Three stages of germination were indicated by the result of cluster analysis. The color criterion is shown on the right. Z and N represent *ZS97* and *NIP*, respectively. The number of 0, 3, 6, 9, 12, 24, 36, and 48 followed N or Z means hours after imbibition. *ZS97*, *Zhenshan97*; *NIP*, *Nipponbare*.

To analyze the dynamic variations in the metabolome between *ZS97* and *NIP* during seed germination, the Pearson correlation coefficient (PCC) of the metabolome was calculated, and cluster analysis was carried out between the two varieties at different time points. The results showed that the metabolomes at eight time-points were divided into three stages (Figure 2). According to the metabolomes of *NIP*, Stage I is from 0 to 12 HAI, Stage II is approximately from 12 to 36 HAI, and Stage III is from 36 to 48 HAI, supporting the three phases of germination indicated by the water absorption rate. Compared with *NIP*, the metabolomic change in *ZS97* was obviously advanced, which was consistent with the germination rate and morphological changes (Figure 1c).

During Stage I, the metabolomes of *ZS97* and *NIP* at 0 HAI were clustered together (Figure 2). Although both varieties showed variations in many traditional agronomic traits, they were nearly identical in the metabolome of dry seeds (PCC = 0.98). At 3 HAI, the metabolomes of both varieties were also very similar (PCC = 0.97), which also showed that there was no obvious change in the metabolome at 3 HAI compared with 0 HAI. Although the water absorption rate changed greatly from 0 to 3 HAI, the morphology of the seeds was basically the same. Then, the metabolomes of both varieties at 6 HAI began to diverge as the PCC dropped to 0.95, while the metabolomes of *NIP* at 6 and 9 HAI were more similar (PCC = 1.00), and the PCC of *NIP* at 3 and 6 HAI reached 0.99. This result indicated that the metabolomes of *NIP* almost did not change from dry seeds to 9 HAI, and the dynamic change in *NIP* in stage I was slow and lagged behind that of *ZS97*. According to the PCC (0.94), between 6 HAI of *ZS97* and 12 HAI of *NIP*, which were clustered together, the lag time for *NIP* was approximately 6 h. During Stage II, the difference in metabolomes between *ZS97* and *NIP* became increasingly obvious. The metabolomes at 9 and 12 HAI for *ZS97* and 24 and 36 HAI for *NIP* were clustered in a single clade, respectively. The metabolome at 24 HAI for *ZS97* was separated from those at 9 and 12 HAI for *ZS97*, showing its advanced metabolic state. Now, the lag time for *NIP* increased to approximately 12 h. During Stage III, the metabolome at 48 HAI for *NIP* was still separated from those at 36 and 48 HAI for *ZS97*, indicating that the lag time was now over 12 h, although both varieties had already completed protrusion of the plumule. Therefore, cluster analysis of metabolomes during germination could properly reflect the process of germination, as predicted by the water absorption rate.

### 2.3. Construction of the Correlation Network between Metabolites and Germination Rate

**Figure 3:**
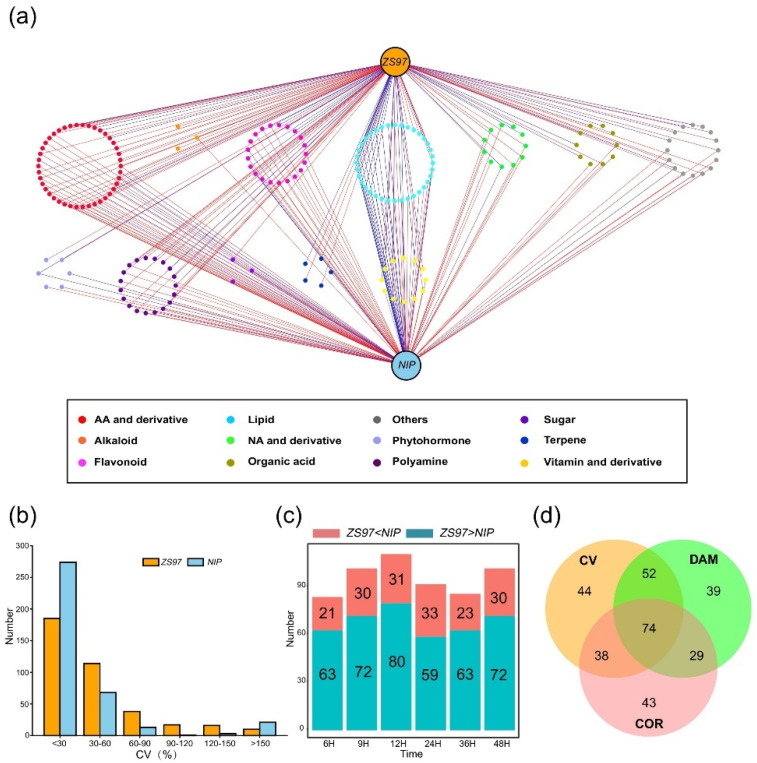
Comparative analysis of metabolites during germination and screening of key metabolites related to germination rate in *ZS97* and *NIP*. (**a**) The correlation analysis results are visualized by Cytoscape. *ZS97* and *NIP* were shown at the top and bottom, respectively. The metabolites significantly related to the germination rate are shown in the middle; each spot represents one metabolite, categories of metabolites are shown in different colors. The line represents a positive (in red) or negative (in blue) correlation between the metabolite and the germination rate. (**b**) Coefficient of variation (CV) analysis results of the metabolome during seed germination of *ZS97* and *NIP*. Numbers of metabolites with different CV were shown. (**c**) The number of metabolites with a difference in content between *ZS97* and *NIP* from 6 HAI to 48 HAI. The relative content of metabolite higher or lower in *ZS97* is shown in blue or red, respectively. H means hours after imbibition. (**d**) The intersection of correlation analysis (COR), coefficient of variation (CV) and differential accumulation of metabolites (DAM), and screening of candidate metabolites closely related to seed germination in rice. *ZS97*, *Zhenshan97*; *NIP*, *Nipponbare*.

To investigate which metabolites specifically accumulated or were consumed during germination, a correlation analysis between the germination rate and metabolite content was performed. In general, if a metabolite is positively related to germination, then it may display a gradual process of accumulation during germination and vice versa. PCC was compared for the two varieties from 6 to 48 HAI, since there was almost no obvious sign of germination or change in the metabolome from 0 to 3 HAI. According to the correlation network acquired, there were 184 significant correlations in total (*p* < 0.05; PCC > |0.8|) (Figure 3a), and the majority of the metabolites associated with the germination rate were lipids, flavonoids, amino acids and their derivatives. The total number of metabolites that were highly correlated with the germination rate in *ZS97* was 128, which was slightly larger than that in *NIP* (123, Table S3). The details of each category of metabolites are shown in Figure S1. It was found that in both *ZS97* and *NIP*, the number of metabolites positively and significantly correlated with the germination rate was highest for amino acids and their derivatives, among which valine and leucine had been previously reported to regulate germination [23,24]. Our study also demonstrated that amino acids and their derivatives may play a crucial role in the physiological process of seed germination. In addition, a large number of lipids were found to be significantly correlated with the germination rate. Most of them were free fatty acids, while a small proportion were phospholipids, and it is conjectured that these metabolites may accumulate and be consumed in response to the energy supply. Moreover, phytohormones were also significantly correlated with the germination rate. For example, salicylic acid (SA) was found to be significantly positively correlated with *ZS97* seed germination (PCC = 0.94, *p*-value = 0.005, Table S3), which was consistent with the results of previous studies [35,36]. The well-known phytohormone ABA was found to be significantly negatively correlated with *NIP* seed germination (PCC = −0.95, *p* = 0.004, Table S3), as reported in numerous studies [15,37].

Therefore, correlation analysis of the dynamic metabolome during germination can help us identify metabolites that affect the germination process. Most of the metabolites positively related to seed germination were amino acids and their derivatives, polyamines, and flavonoids. Most of the metabolites negatively related to seed germination were lipids, other kinds of flavonoids, and amino acids and their derivatives.

### 2.4. Identification of Unique and Differentially Accumulated Metabolites between the Two Varieties

If a metabolite plays a regulatory role in seed germination, then its content normally fluctuates during the whole process to adapt to the physiological changes of the seed. Hence, the coefficient of variation (CV) was used to measure the discrete degree of variation in metabolite levels during seed germination (Figure 3b). The results showed that the CV of most metabolites in *ZS97* and *NIP* was less than 30%, indicating that the content of most metabolites in the two varieties did not fluctuate greatly and that they may not have a strong relationship with seed germination. In contrast, metabolites with higher CV values were considered to have fluctuating levels and to be more likely to influence seed germination progress. According to the threshold of over 30%, 190 and 104 metabolites were identified in *ZS97* and *NIP*, respectively (Figure 3b, Table S1).

Since the metabolomes of *ZS97* and *NIP* began to diverge at 6 HAI, an analysis of the differential accumulation of metabolites (DAM) between the two varieties was performed at six time-points from 6 to 48 HAI. Similar to the CV results, only a small number of metabolites were different between the two varieties, and it was possible that these different metabolites were the cause or result of the different germination speeds between the two varieties. Then, the contents of metabolites in the two varieties were compared. Statistically, there were 84 metabolites with different content (*ZS97* > *NIP* or *ZS97* < *NIP*) at 6 HAI between the two varieties, and that number fluctuated to a certain degree as germination progressed (Figure 3c).

### 2.5. Screening of Candidate Metabolites Affecting Rice Germination

To date, three methods, COR, CV, and DAM, have been used to detect possible metabolites related to the germination rate for two varieties studied here, and a set of metabolites has been obtained by each method. Of course, some of these metabolites may not truly be related to seed germination. To identify the key metabolites that might truly affect germination, the intersection of three sets of data was extracted and 74 candidate metabolites were acquired (Figure 3d, Table S4). Afterwards, these screened metabolites were classified. The results showed that most of them were amino acids and their derivatives (16), followed by polyamines (12), lipids (9), flavonoids (7), organic acids (6), terpenes (5), nucleotides and derivatives (3), phytohormones (2), sugars (2), vitamins and derivatives (2), and others (10). This result suggested that amino acids and their derivatives may have a vital influence on germination, as expected since they are involved in almost all growth and development processes as primary metabolites [23,25]. The function of polyamines in seed germination has been reported in many studies [17,20,21]. Seed germination is an energy-demanding process, and sugars and lipids are the main energy substances in seed germination, so their influence on germination is also crucial. However, in this study, the metabolites were determined by HPLC–MS/MS, which resulted in fewer sugars being detected. Flavonoids have been relatively less studied in seed germination until now, but they have antioxidant effects, and the plant seed germination process is sensitive to oxidation, so it is possible that flavonoids may affect seed germination by antioxidation.

**Figure 4:**
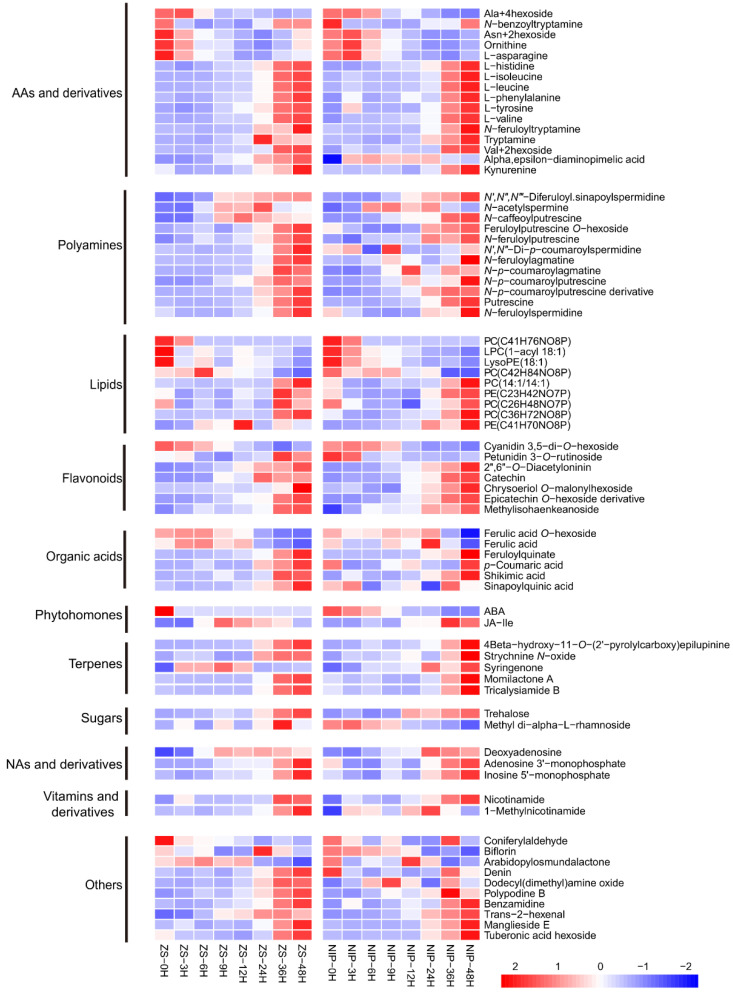
Accumulation/consumption patterns of screened candidate metabolites affecting germination rate in two varieties of *ZS97* and *NIP*. Categories of metabolites are shown on the left. AAs, amino acids; NAs, nucleotides. The relative content of each metabolite in different stages of germination was standardized and shown in the box according to the color criterion (Z-score). The name of each metabolite was annotated on the right. 0, 3, 6, 9, 12, 24, 36, and 48 h means hours after imbibition. ZS, *ZS97*; *Zhenshan97*; NIP, *Nipponbare*.

Subsequently, the accumulation/consumption patterns of these 74 candidate metabolites during germination were analyzed (Figure 4). Overall, most of these metabolites showed an accumulation pattern, and only a few metabolites showed a consumption pattern during germination. Among these candidates, the phytohormone ABA, a well-known inhibitor of seed germination, was included as expected; it showed an overall consumption pattern during germination, which was also consistent with its inhibitory role in germination. In *ZS97*, the ABA content was high at 0 HAI, but it decreased quickly to a low level at 3 HAI that was maintained during the later processes. For *NIP*, the level of ABA at 0 HAI was not as high as that of *ZS97*, but it decreased more slowly, and the content was still relatively higher at 3 and 6 HAI. The difference in the ABA consumption speed may contribute to the difference in the germination speed between the two varieties. It is worth noting that several amino acids, including leucine, isoleucine, phenylalanine, and valine, showed a trend of accumulation during germination, as reported by a previous study [23]. Gipson et al. (2017) found that inhibiting valine degradation can affect the development and germination of Arabidopsis seeds, indicating that maintaining its low level in the early germination stage is important [24]. In addition, polyamine has been reported frequently for its promotion effect on germination [17,20,21,38]. There were twelve members of the polyamine family included in the 74 candidates, and as expected, they showed a pattern of accumulation during germination in both varieties (N-acetylspermine is special with a pattern of accumulation before consumption). These results suggested that the screening analysis in our study was effective and reliable. Except for a few metabolites, such as ABA, valine, and asparagine, which have been studied previously [14,24,39], the function of other metabolites during germination deserve special attention in the future.

### 2.6. Elucidation of Two Metabolic Modules among the Screened Candidates and Verification of Shikimic Acid’s Effect on Seed Germination

**Figure 5:**
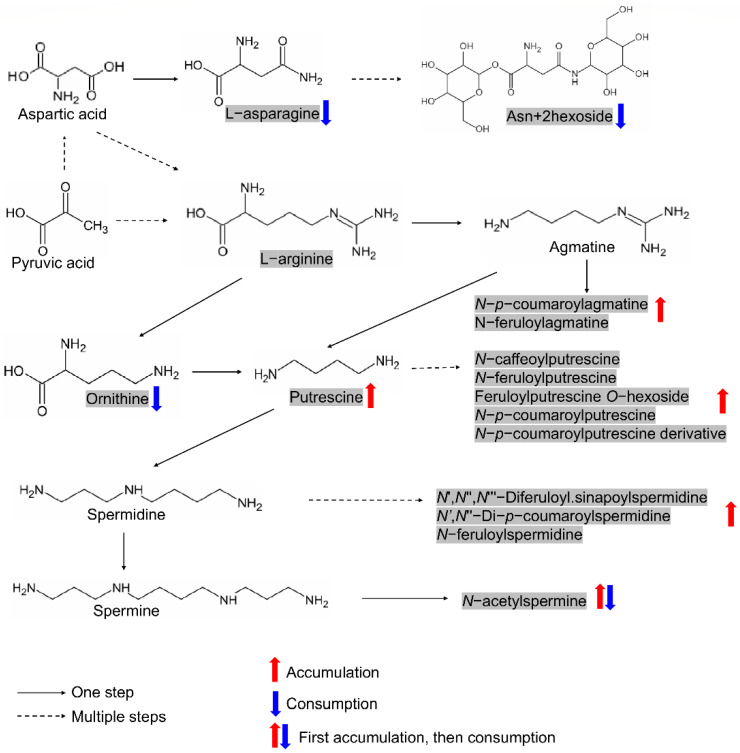
The ornithine–asparagine–polyamine module formed by metabolites originated from nitrogen-rich amino acids/derivatives. The metabolites in shadow represent candidates screened in this study. Metabolites with consistent accumulation or consumption patterns during germination in both varieties were marked with red or blue arrows, respectively.

**Figure 6:**
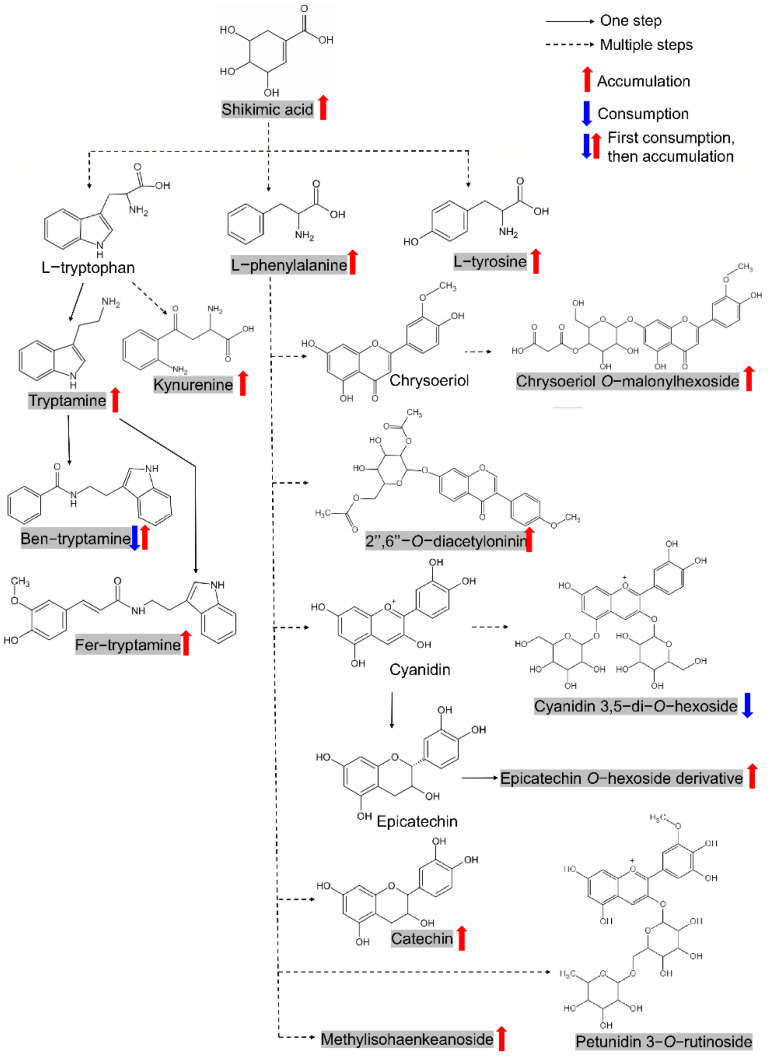
The shikimic acid–tyrosine–tryptamine–phenylalanine–flavonoid module formed by metabolites originated from aromatic amino acids/derivatives. The metabolites in shadow represent candidates screened in this study. Metabolites with consistent accumulation or consumption patterns during germination in both varieties were marked with red or blue arrows, respectively. Ben-tryptamine, *N*-benzoyltryptamine; Fer-tryptamine, *N*-feruloyltryptamine. The structure of epicatechin *O*-hexoside derivative is not shown due to the undetermined modifying group. The structure of Methylisohaenkeanoside is not shown due to the complexity (refer: https://www.chem960.com/structure/sc119864050/ (accessed on 14 December 2021)).

Since amino acids and their derivatives are most abundant in the 74 screened candidates, we analyzed them one by one and found that they included three distinctive groups of metabolites. The first group is branched-chain amino acids/derivatives (L-valine, L-leucine, L-isoleucine, and Val + 2hexoside). The second one is aromatic amino acids/derivatives (L-phenylalanine, L-tyrosine, and four L-tryptophan derivatives), and the third one is nitrogen-rich amino acids/derivatives (L-asparagine, Asn + 2hexoside, and ornithine). Of course, branched-chain amino acids’ role in seed germination has been reported previously [23,24]. The aromatic and nitrogen-rich amino acids/derivatives were found here to act with other metabolites to form two metabolic modules that include a total of 29 metabolites. One was the ornithine–asparagine–polyamine module (Figure 5), and the other was the shikimic acid–tyrosine–tryptamine–phenylalanine–flavonoid module (Figure 6).

The promotive role of polyamine in seed germination has been reported extensively [17,20,21,38]. As nitrogen-rich amino acids, ornithine, and arginine are important precursors of nitrogen-containing substances in living organisms since they can be decarboxylated to form polyamines. In this study, only ornithine but not arginine was screened as the candidate metabolite, although arginine’s function in seed germination has also been extensively accepted [40,41]. However, we screened one amino acid derivative, asparagine, whose involvement in the nitrogen flow by interconversion with arginine during seed formation and germination has been reported in cotton [42]. Asparagine was also found to accumulate in dry seeds and was the predominant amide throughout germination in sunflowers [39]. These studies suggest that asparagine may also be involved in the nitrogen flow during seed germination and participates to form the ornithine–asparagine–polyamine module (Figure 5). Of 74 metabolites screened, 15 metabolites belonged to this module, including ornithine, L-asparagine, Asn + 2hexoside and twelve polyamines (putrescine/spermidine/agmatine and their derivatives). As expected, both putrescine and spermidine, along with their derivatives, showed accumulation patterns (*N*-acetylspermine is special), while ornithine, L-asparagine, and Asn + 2hexoside showed consumption patterns during rice germination according to our results (Figure 5). Therefore, the involvement of the ornithine–asparagine–polyamine module in affecting seed germination in rice is reasonable.

The shikimate pathway plays a central role in plant secondary metabolic pathways since it directs bulk carbon flow towards the biosynthesis of aromatic amino acids (tyrosine, phenylalanine, and tryptophan) and numerous aromatic phytochemicals, including flavonoids [43]. Shikimate (shikimic acid) itself can even play a regulatory role in plant phenylalanine metabolism [44]. Of the 74 metabolites screened in our study, 14 metabolites belonged to this pathway, including shikimic acid, tyrosine, tryptophan derivatives (tryptamine, *N*-benzoyltryptamine, *N*-feruloyltryptamine, and kynurenine), phenylalanine and seven flavonoids, thus forming the shikimic acid–tyrosine–tryptamine–phenylalanine–flavonoid module (Figure 6). Interestingly, most members of this module show an accumulation pattern during germination, except for cyanidin 3,5−di−*O*−hexoside, possibly indicating this module’s positive role in seed germination. In fact, the accumulation of tryptamine in seed germination has been reported in legumes [45]. Flavonoids, which perform an enormous range of biological functions in plants, were also reported to affect seed germination, possibly due to the significant enhancement of antioxidant activities [32,46].

**Figure 7:**
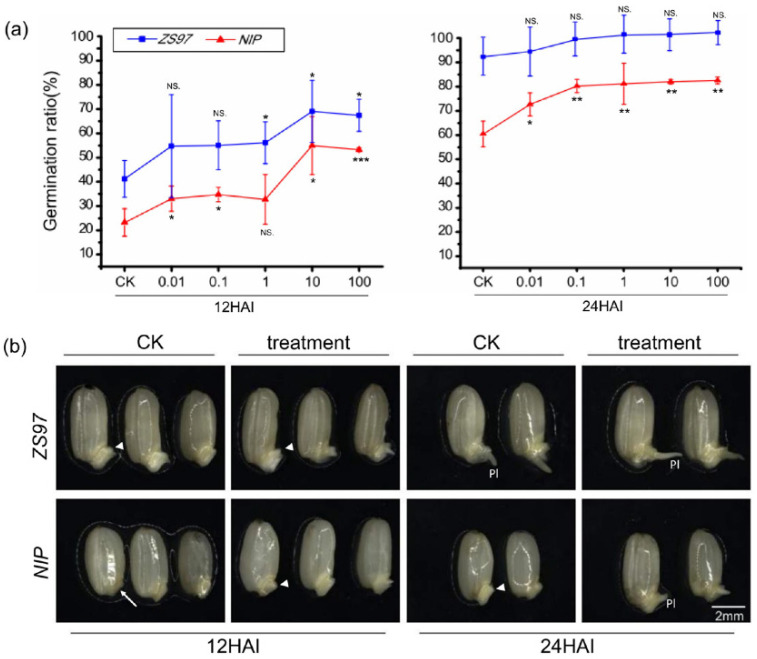
Effect of exogenous shikimic acid treatment on the germination process and morphology of seeds for *ZS97* and *NIP*. (**a**) Effect of exogenous shikimic acid on the germination process. Statistics analysis was performed for 12 and 24 HAI. Different concentrations of shikimic acid were shown in X-axis, the germination rate was shown on the Y-axis. Results of Student’s *t*-test (compare different time points with control, CK): * *p* < 0.05; ** *p* < 0.01; *** *p* < 0.001. NS, no significant difference. (**b**) Effect of shikimic acid on morphological changes of seeds. Treatments without (CK) or with 1 μg/L shikimic acid (treatment) are shown. The embryo is indicated by the arrow. The emergence of plumule is indicated by the arrowhead. Pl, plumule. HAI, hours after imbibition. The bar is shown as 2 mm. *ZS97*, *Zhenshan97*; *NIP*, *Nipponbare*.

As the core member of the shikimate pathway, which directly affects the formation of many aromatic compounds, shikimic acid has attracted much attention for its well-known defense function in plants, while its function in seed germination has not been revealed. Therefore, exogenous treatment with shikimic acid was carried out in this study to verify its effect on seed germination. A series of concentration gradients (0.01, 0.1, 1, 10, and 100 μg/L) of shikimic acid were used, and the germination rates at 12 and 24 HAI were statistically analyzed in *ZS97* and *NIP*. The results showed that shikimic acid significantly promoted seed germination in both varieties (although the effect is not significant statistically at 24HAI in *ZS97*, whose germination rate of CK is very high) (Figure 7a). With increasing concentration (especially at 1, 10, and 100 μg/L), the effect of shikimic acid on seed germination became increasingly significant. Morphological observations also supported shikimic acid’s role in promoting seed germination (Figure 7b, the result for 1 μg/L shikimic acid is shown). Thus, it is reasonable to conclude that shikimic acid is a promoter of seed germination.

## 3. Discussion

Seed germination is an important stage that determines whether a seed will successfully grow into a seedling. At the physiological level, germination is a process of seeds changing from a heterotrophic to an autotrophic state, and metabolites undoubtedly play essential roles during this process since they contribute the raw materials for cellular structure assembly, energy supply, biochemical reactions, and signaling, which are all necessary for the completion of seed germination [5,6,7]. Nevertheless, the elucidation of key metabolites/metabolic pathways that regulate seed germination is still limited, although the functions of some metabolites have been clarified [14,21,24]. Therefore, revealing the metabolites affecting seed germination and establishing the metabolic network are still primary tasks for biologists and agricultural scientists in this field.

### 3.1. Metabolomics Is an Effective and Meaningful Way to Explore the Mechanism of Seed Germination

Since there is a complex network of relationships among metabolites in plants, the detection of metabolites individually is time-consuming and makes it difficult to reveal complex metabolic processes. At the same time, the metabolic changes during seed germination are very rapid, especially in the early stage of germination [33]. Therefore, efficient detection of metabolites will contribute to the elucidation of the mechanism underlying seed germination.

As an important branch of systematic biology, metabolomics has received widespread attention in the field of plant research in recent years, and a series of important advances have been made, especially regarding the regulatory mechanisms of some traditional agronomic traits [47,48]. Metabolomics has also been frequently used in the study of seed germination in recent years [26,27,28,49]. In Arabidopsis, metabolic and transcriptomic correlation analysis identified two distinctive profiles involved in the metabolic preparation for seed germination and seedling establishment, respectively [12]. In barley, the changes of metabolite profiles and starch-degrading enzymes during grain germination, and as affected by GA and abscisic acid (ABA) were investigated using two wild barley accessions XZ72 and XZ95; results showed that the change of metabolite during germination was time- and genotype-dependent, and addition of GA enhanced the activities of starch-degrading enzymes, and increased most metabolites, especially sugars and amino acids, whereas ABA had the opposite effect [50]. The metabolic distributions of germinating seeds from two different inbreds of maize (*Zea mays*) seeds, B73 and Mo17, showed that the two inbreds are highly differentiated in their metabolite profiles throughout the course of germination, especially with regard to amino acids, sugar alcohols, and small organic acids [49]. In this study, we investigated the metabolome of germinating seeds at different time points from two varieties of rice with comparative analysis. It was found that the metabolomes of seeds can properly reflect the process of germination and even the differences between varieties with different germination speeds, indicating the great application value of metabolomics in the detection of biological processes. By using two rice varieties with different germination speeds, we carried out a comparative analysis and finally acquired 74 candidate metabolites putatively related to seed germination, which laid a foundation to further reveal the mechanism of seed germination in rice.

Therefore, metabolomics can help researchers effectively detect the accumulation or consumption of metabolites, which is helpful for revealing the dynamic physiological processes involved in seed germination. Furthermore, the phenotypic changes in morphology during early seed germination are relatively small, but metabolomics can be used to observe changes happening during early germination and to explore which metabolites or metabolic pathways can regulate seed germination.

### 3.2. The Regulation of Metabolic Modules on Seed Germination Is Worthy of Attention

There are some well-known metabolites that have been reported to affect seed germination, such as ABA, GA, polyamine, and some amino acids and their derivatives [14,22,24]. These metabolites pave the way for studying the mechanism of seed germination by metabolomics. By using three typical methods, COR, CV, and DAM, for comparative metabolome analysis at different time points in two rice varieties with different germination rates in this study, we identified 74 candidate metabolites associated with germination, including the well-known molecules ABA, valine, and polyamines. Although we did not directly prove the regulatory function of these candidate metabolites on seed germination individually, we found two metabolic modules that are potentially crucial for seed germination in rice. One is the ornithine–asparagine–polyamine module, and the other is the shikimic acid–tyrosine–tryptamine–phenylalanine–flavonoid module. Most of the metabolites in both modules have been previously reported in different species to be involved in seed germination. This result suggests the reliability of our screening results and helps clarify the regulatory mechanism of seed germination in rice.

The ornithine–asparagine–polyamine module is closely related to the pathway of arginine and proline metabolism, in other words, nitrogen flow, which is essential for seed germination. Polyamines usually act as developmental regulators and play key roles in numerous physiological processes, including seed germination [22]. During germination, the levels of polyamines in soybean were found to increase significantly, with spermidine and spermine accumulating in the cotyledon and cadaverine and putrescine accumulating in the radicle and hypocotyl [51]. Nitric oxide–polyamine cross-talk may even exist during the dormancy release and germination of apple embryos [38]. Exogenous treatment with spermidine was found to improve seed germination via its involvement in phytohormone interactions, antioxidant defenses and relevant gene expression [20,21]. Ornithine and arginine are both nitrogenous amino acids that can form polyamines through decarboxylation. Llebres et al. (2018) identified the genes involved in arginine metabolism in *Pinus pinaster* Ait and found that arginine plays an important role in seed germination [41]. Ornithine may affect seed germination through arginine metabolism or metabolic conversion to arginine by sequential activities of enzymes ornithine transcarbamylase, argininosuccinate synthetase, and argininosuccinate lyase [40,52,53]. In cotton, nitrogen flow occurs in seed formation and germination through an asparagine cycle involving the following sequence: asparagine —> arginine —> storage protein —> arginine —> asparagine [42]. Therefore, we are not surprised that asparagine and arginine were frequently found together during seed germination [25,54]. Therefore, ornithine, arginine, asparagine, and polyamine are closely related, and it is reasonable to hypothesize that these metabolites may regulate seed germination in rice through cooperation. Confirmation and detailed analysis of this module’s regulation of rice seed germination needs to be further investigated.

The shikimic acid–tyrosine–tryptamine–phenylalanine–flavonoid module is closely related to the pathway of phenylalanine, tyrosine and tryptophan biosynthesis along with biosynthesis of phenylpropanoids. This module may be more well known for its involvement in defense, especially shikimic acid (shikimate). Some of the members of this pathway have been reported to be involved in seed germination. In legume seeds, tryptamine was found to be the main biogenic amine detected, and its concentration considerably increased during the germination process [45]. Flavonoids are common polyphenol secondary metabolites that exist in various organs of plants and have a wide range of regulatory effects on plant growth and development. By comprehensive profiling and natural variation analysis of flavonoids in rice, it was found that a number of major flavonoids accumulated in germinated seeds [55]. Flavonoid biosynthesis may be involved in mediating the differences in germination speed and young seedling growth between two types of rice [32]. Of course, due to the large diversity of flavonoids, their function in seed germination is complicated. For example, in warm-season grasses, catechin acted synergistically with the nitric oxide donor sodium nitroprusside and nitrite to promote seed germination [56]. Naringenin was found to inhibit seed germination and seedling root growth through a salicylic acid-independent mechanism in *Arabidopsis thaliana* [46]. As the core molecule of the upstream pathway in this module, shikimic acid’s involvement in seed germination has been reported, although its direct function was not detected. It was found that presoaking seeds with shikimic acid can improve the growth, productivity, and quality of tomato plants [57]. Ellagic acid treatment accelerated the germination and seedling growth of chickpea under osmotic stress conditions by reducing the levels of hydrogen peroxide and increasing antioxidant capacity with an increase in flavonoids, enzymes of the shikimic acid pathway, and the activity of antioxidant enzymes [58]. Our exogenous treatment results here directly prove shikimic acid’s regulation of seed germination, further supporting the involvement of the shikimic acid–tyrosine–tryptamine–phenylalanine–flavonoid module in regulating seed germination.

So, during seed germination, a metabolic system with drastic adjustment in physiology, the two modules elucidated here might meet the demand on nitrogen flow and antioxidant and thus have a role in guaranteeing the completion of it. Of course, we need to further explore the molecular mechanism of both modules in regulating seed germination in rice. And other metabolites included in 74 candidates also need to be analyzed to verify their role in seed germination. For example, lipids were prominent in our study, but their exact functions in seed germination are still unclear. For vitamins, accumulation of vitamin C, folate and α-tocopherol has been reported in germinating seeds [59,60,61]. We found here that the nicotinamide and 1-methylnicotinamide might also be key metabolites closely related to seed germination in rice. In fact, nicotinamidase activity was found to be important for germination, which involves NAD-degrading poly (ADP-ribose) polymerases (PARP enzymes) activity and DNA repair responses in Arabidopsis [62]. So, the regulation of nicotinamide homeostasis is worthy of attention for study in seed germination. 

Another issue deserved to explore is the key enzymes related to metabolic pathways involved in seed germination. Just as the cytosolic fructose-1,6-bisphosphatase (cFBPase) from mung beans, which acts as a rate-limiting enzyme in gluconeogenesis, separation and structural analysis of it is meaningful for precise crop breeding and food chemistry [63]. The key enzymes related to the ornithine–asparagine–polyamine module here include arginine decarboxylase, agmatinase, arginase, and ornithine decarboxylase et al. [64], and the key enzymes related to the shikimic acid–tyrosine–tryptamine–phenylalanine–flavonoid module here include shikimate kinase, chorismate mutase, aromatic-amino-acid transaminase, phenylalanine ammonia-lyase, cinnamic acid 4 hydroxylase, and hydroxycinnamoyl-CoA shikimate/quinate hydroxycinnamoyl transferase et al. [44,65]. They are all potential targets to operate in the agricultural research and food industry. 

## 4. Materials and Methods

### 4.1. Material

Two typical rice (*Oryza sativa*) varieties, *Zhenshan 97* (*ZS97*, *Indica*) and *Nipponbare* (*NIP*, *Japonica*), were used in this study. Both lines were planted in Wuhan, harvested and preserved when they were mature and with healthy seeds.

### 4.2. Seed Germination Rate Statistical Method

The rice seeds stored in the seed cabinet were taken out and baked at 37 °C for 3 days before the germination experiment. For each variety to germinate, seeds were inoculated with three petri dishes (diameter 9 cm), 50 shelled seeds were incubated in each petri dish with filter paper laid in advance and moistened with 5 mL single sterilized water. Incubation was carried out for 48 h with 16/8 h of light/dark at 28 °C. Since the change in morphology in the early germination stage is not obvious to the naked eye, seed germination at different time points (0, 3, 6, 9, 12, 24, 36, and 48 HAI) was observed under a stereomicroscope (LEICA M205 FA) for amplification. The petri dish with germinating seeds was put directly under the stereomicroscope for observation. The criterion for germination is that the seed coat is broken and plumule or radicle is exposed. 

### 4.3. Water Absorption Rate Statistical Method

To calculate the water absorption rate, ten seeds were taken at each time point, and the surface moisture was wiped dry for weighing. Then the seeds were placed in a 60 °C oven for three days to dry. The ratio of the difference between the weight before (fresh weight, W_a_) and after (dry weight, W_b_) drying to the dry weight is the water absorption rate (A_r_). The formula is A_r_ = (W_a_ − W_b_)/W_b_ × 100%. Three biological replicates were done for each variety. 

### 4.4. Extraction and Detection of Metabolites

Sample Preparation and Extraction: Seed samples were extracted as described previously [66]. In brief, the seeds at different time points of germination were first freeze-dried and then ground into powder using a mixer mill (MM400, Retsch, Hahn, Germany) for 1.5 min at 30 Hz. 100 mg powder was weighed and extracted overnight at 4 °C with 1 mL 70% aqueous methanol, followed by centrifugation for 10 min at 13,000× *g*. The supernatants were filtrated (SCAA-104, 0.22 mm pore size; ANPEL Shanghai, China) before LC–MS analysis.

Metabolome analysis: For metabolome analysis, samples were analyzed using a high-performance liquid chromatography (HPLC)-based targeted method combined with a nontargeted metabolic profiling method [66,67,68,69]. Nontargeted metabolites profiling analysis was performed by using a HPLC–MS/MS system (HPLC, UltiMate3000; MS, Q Exactive Plus MS system, Thermo Fisher Scientific, Waltham, MA, USA). The analytical conditions were as follows, HPLC: column, shim-pack GISS C18 (pore size 1.9 μm, length 2.1 × 100 mm); solvent system, water (0.04% acetic acid): acetonitrile (0.04% acetic acid); gradient program, 0 min, 5%B; 13.5 min, 95%B; 15.0 min, 95%B; 15.1 min, 5%B; 17 min, 5%B; flow rate, 0.4 mL/min; temperature, 40 °C; injection volume: 2 μL. Mass spectrometric detection used HESI ion source for nontargeted metabolites profiling in Full MS and ddMS2 mode to obtain the data, including the accurate masses, MS/MS fragments, and retention times. The recording conditions were as followings: source capillary, 3.2 kV (positive); sheath gas flow rate, 7 psi; source temperature, 400 °C; scan range, m/z 100–1500 [66]. The targeted metabolic profiling analysis was done by using a scheduled multiple reaction monitoring (MRM) via LC-ESI-QQQ-MS/MS system (Applied Biosystems 4000 Q TRAP, AB Sciex, USA) with ESI Turbo Ionspray interface and controlled by Analyst 1.6 software. The ESI source operation parameters were as follows: source temperature, 500 °C; ion spray voltage (IS), 5500 V; ion source gas I (GSI); gas II (GSII), and curtain gas (CUR) were set at 55, 60, and 25.0 psi, respectively; the collision gas (CAD) was high. The LC condition was the same as described above. To produce a maximal signal, collision energy (CE) and declustering potential (DP) were optimized for each precursor-product ion (Q1–Q3) transition [67,68,69] (Figure S2).

### 4.5. Statistical Analysis

The content of metabolite and germination rate were used to calculate the correlation coefficient. The R script function cor. test with the Pearson method was used to determine the correlation coefficient, and the network diagram was displayed through the software Cytoscape 3.7.1. The differential analysis of metabolite was performed based on the average content of three biological replicates at the same time point, and the significance test was performed using the student’s test. The difference fold change is more than two times and the *p*–value was less than 0.05 was considered as a significant difference. And the *p*-value was adjusted using Benjaminiand–Hochberg method under multiple testing correction. R package “pheatmap” was used to cluster and display metabolomes. The coefficient of variation (CV) analysis was performed by the formula C.V. = σ/μ and the data of each metabolite content was used.

### 4.6. Exogenous Treatment with Shikimic Acid

A series of concentration gradients as 0, 0.1, 1, 10, and 100 μg/L were set for shikimic acid to treat the seeds. 5 mL single sterilized water with different concentrations of shikimic acid were added to each petri dish at the beginning. Germination rates at two time-points (12 and 24HAI) were counted.

## Data Availability

The data presented in this study are openly available in article.

## Supplementary Materials

The following are available online at https://www.mdpi.com/article/10.3390/metabo11120880/s1, Figure S1: Statistics of each category of metabolites significantly related to germination rate in *ZS97* and *NIP*, Figure S2: Identification and annotation of metabolites based on high-resolution MS2 data, Table S1: Scheduled multiple reaction monitoring transitions for widely targeted metabolite analysis in this study and result of coefficient of variation analysis, Table S2: Raw data about the relative content of 380 metabolites detected in two varieties, Table S3: Result of correlation analysis between germination rate and metabolite content with Pearson correlation coefficient in two varieties, Table S4: List of 74 screened candidates closely related to seed germination in rice.
