# Supplementary material for: Comparative Metabolomics Reveals Two Metabolic Modules Affecting Seed Germination in Rice (Oryza sativa)
Source: Metabolites. 2021 Dec 17;11(12):880. doi: 10.3390/metabo11120880 (PMC8707830; doi:10.3390/metabo11120880)
Supplement: Supplementary file 1 [file metabolites-11-00880-s001.zip › metabolites-1470145-supplementary/Supplementary Files/Supplementary Figures.pdf]

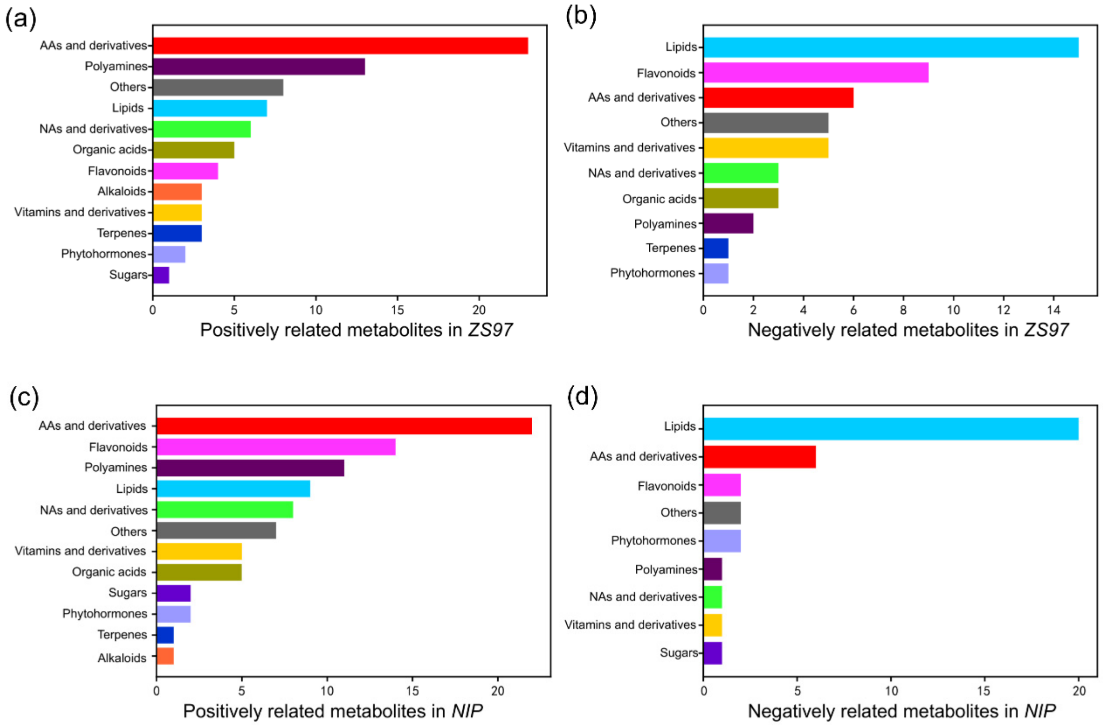

Figure S1. Statistics of each category of metabolites significantly related to germination rate in *ZS97* and *NIP*. (a) and (c) Statistics of metabolites positively correlated with germination rate in *ZS97* and *NIP*, respectively (PCC>0.8, p value<0.05). (b) and (d) Statistics of metabolites negatively correlated with germination rate in *ZS97* and *NIP* respectively (PCC<-0.8, p value<0.05). The categories of metabolites were shown in Y-axis. The number of metabolites for each category is shown in X-axis. AAs, amino acids; NAs, nucleotides. ZS97, *Zhenshan97*. NIP, *Nipponbare*.

(a)

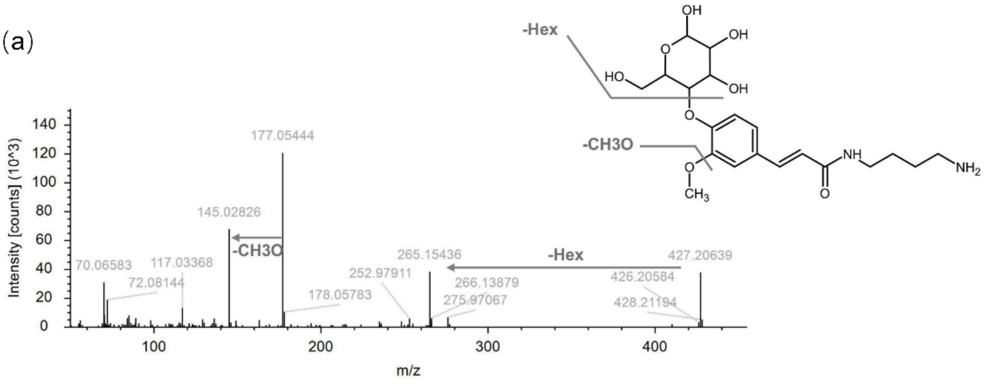

(b)

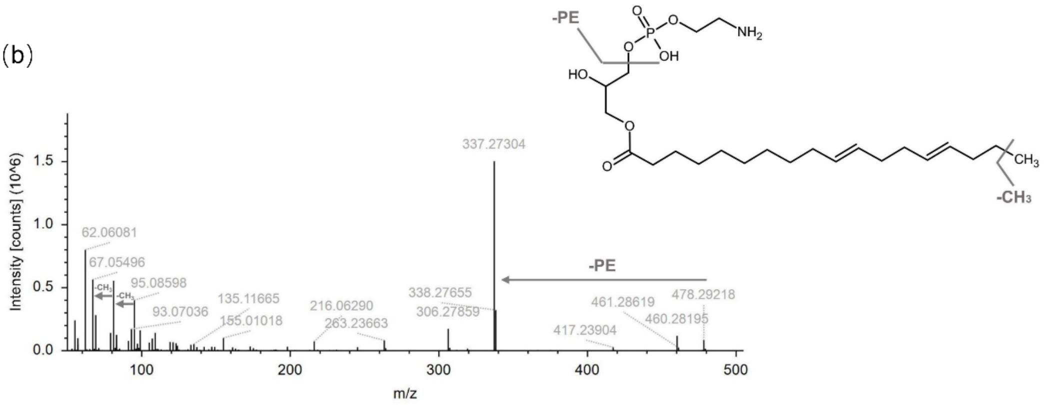

Figure S2. Identification and annotation of metabolites based on high-resolution MS2 data. (a) Feruloylputrescine O-hexoside. (b) LysoPE (18:2). The arrows in the MS2 plots indicate neutral loss of metabolites, and the corresponding positions are marked in the substance structure diagrams.
